# Variation in Bacterial Community Structure Under Long-Term Fertilization, Tillage, and Cover Cropping in Continuous Cotton Production

**DOI:** 10.3389/fmicb.2022.847005

**Published:** 2022-04-04

**Authors:** Ning Duan, Lidong Li, Xiaolong Liang, Aubrey Fine, Jie Zhuang, Mark Radosevich, Sean M. Schaeffer

**Affiliations:** ^1^Department of Biosystems Engineering and Soil Science, The University of Tennessee, Knoxville, Knoxville, TN, United States; ^2^Department of Agronomy and Horticulture, University of Nebraska–Lincoln, Lincoln, NE, United States; ^3^Department of Earth and Planetary Sciences, Washington University in St. Louis, St. Louis, MO, United States; ^4^Center for Environmental Biotechnology, The University of Tennessee, Knoxville, Knoxville, TN, United States

**Keywords:** soil bacterial diversity, 16S rRNA, agricultural management practices, N fixer, nitrifier, denitrifier

## Abstract

Agricultural practices alter the structure and functions of soil microbial community. However, few studies have documented the alterations of bacterial communities in soils under long-term conservation management practices for continuous crop production. In this study, we evaluated soil bacterial diversity using 16S rRNA gene sequencing and soil physical and chemical properties within 12 combinations of inorganic N fertilization, cover cropping, and tillage throughout a cotton production cycle. Soil was collected from field plots of the West Tennessee Agriculture Research and Education Center in Jackson, TN, United States. The site has been under continuous cotton production for 38 years. A total of 38,038 OTUs were detected across 171 soil samples. The dominant bacterial phyla were *Proteobacteria*, *Acidobacteria*, *Actinobacteria*, *Verrucomicrobia*, and *Chloroflexi*, accounting for ∼70% of the total bacterial community membership. Conventional tillage increased alpha diversity in soil samples collected in different stages of cotton production. The effects of inorganic N fertilization and conventional tillage on the structure of bacterial communities were significant at all four sampling dates (*p* < 0.01). However, cover cropping (*p* < 0.05) and soil moisture content (*p* < 0.05) only showed significant influence on the bacterial community structure after burn-down of the cover crops and before planting of cotton (May). Nitrate-N appeared to have a significant effect on the structure of bacterial communities after inorganic fertilization and at the peak of cotton growth (*p* < 0.01). Structural equation modeling revealed that the relative abundances of denitrifying and nitrifying bacteria were higher when conventional tillage and vetch cover crop practices were applied, respectively. Our results indicate that long-term tillage and fertilization are key factors increasing the diversity and restructuring the composition of bacterial communities, whereas cover cropping may have shorter-term effects on soil bacteria community structure. In this study, management practices might positively influence relative abundances of bacterial functional groups associated with N cycling. The bacteria functional groups may build a network for providing N and meet microbial N needs in the long term.

## Introduction

Soil microorganism are decomposers and nutrient transformers and promote various biogeochemical cycles (BGC) through redox reactions (Timonen et al., 1996; Trevors, 1998; Kibblewhite et al., 2008; Meliani et al., 2012). They perform critical ecological roles in agricultural systems such as C and N cycling (Aczel, 2019; Tian et al., 2019; Basu et al., 2021; Prasad et al., 2021). Microbial taxa can be involved in specific process of nutrient transformation, such as lignin decomposition, N fixation, nitrification, and denitrification associated with these C and N transformation processes (Anand et al., 2015; Jiménez-Bueno et al., 2016; Liu et al., 2020). For example, the bacterial strain in *Streptomyces viridosporus* is able to secret extracellular oxidative enzymes to break down lignin and provide substrate for other microbes and plant to uptake (Ramachandra et al., 1988; Brown and Chang, 2014). *Paenibacillus* strains with N-fixing capability have a positive effect on crop yield and root growth, and *Rhizobium* can promote the efficient N fixation cooperatively with legumes (Etemadi et al., 2018; Liu et al., 2019).

Changes in the soil microbiome over 40 years of cotton planting remains unclear (Xi et al., 2019). Few studies of studying microbial community composition and structure on our study site are reported (Hu et al., 2021a; Li et al., 2021). Long-term agricultural management practices, such as tillage, inorganic N fertilization, and cover cropping system, can influence the structure and functions of soil bacterial communities (Rousk et al., 2009, 2010; Ramirez et al., 2010; Geisseler and Scow, 2014; Zhou et al., 2017; Dai et al., 2018; Zhang C. et al., 2019; Hu et al., 2021a). The effects of management practices on N inputs not only manifest, in turn, as increases in crop growth and soil organic matter inputs but also can lead to increasing soil acidity with concomitant decreases in diversity of bacteria. Previous study shows that microbial organism structure has a particular change on composition of microbial community involved in N cycling after N fertilizer application (Gao et al., 2020). For example, inorganic N fertilization can significantly impact *Nitrosospira*, ammonia oxidizing bacteria, which were dominant in soils with N fertilizer addition (Bruns et al., 1999; Wu et al., 2011; Liu et al., 2018; Hu et al., 2021b).

Tillage management increases soil aeration and the degree of mixing of crop residues and decreases soil aggregation and water infiltration, thereby altering microenvironments and impacting microbial community structure through bottom-up environmental controls (Kladivko, 2001; Lienhard et al., 2013). Bacterial diversity tends to decrease under intensive tillage, whereas reduced or no tillage can increase bacterial diversity due to creation/preservation of microhabitats at the pore-scale and alterations of the organic matter decomposition rates in contrasting tillage systems (Wang et al., 2017; Legrand et al., 2018). However, no-tillage can drive a decrease of soil porosity and increase soil bulk density, leading to soil compaction compared to regularly tilled systems (Logsdon and Karlen, 2004; Pastorelli et al., 2013), influencing water and oxygen diffusions and creating anaerobic environments favorable to the growth of denitrifiers (Tatti et al., 2015). Crop residues retention favored from zero-tillage would create a suitable environment for organic matter decomposers, for example, *Actinobacteria* are more abundant in zero-tillage systems (Navarro-Noya et al., 2013; Jiménez-Bueno et al., 2016).

Cover cropping increases soil organic matter input and protects soils from erosion and nutrient losses through leaching and runoff (Romdhane et al., 2019). In addition, soil surfaces covered by cover crop residue can reduce soil temperature fluctuation and water evaporation. The growth of genera of *Bacillus* and *Pseudomonas* can be benefit from cover cropping (Patkowska and Konopiński, 2013). Root exudates contain carbon-rich substrate (e.g., amino acids, organic acids, sugars, and phenolics), which attract a variety of rhizosphere microbes including N-fixing bacteria (Rasmann et al., 2005; Vukicevich et al., 2016; Finney et al., 2017). Crop and root exudates positively influence soil microbial biomass and activity (Bardgett and Shine, 1999; Bending et al., 2002; Mbuthia, 2014; Vukicevich et al., 2016; Finney et al., 2017). The abundances of nitrifiers and nitrification processes can increase under the combination of crops and N fertilizer (Li H. et al., 2019). Therefore, cover cropping has a potential to alter soil bacterial communities and functional taxa.

N cycling is the key component in BGCs of agricultural soil systems, and nitrogen availability restricts the plant production, organic matter input, and soil fertility (Greenwood, 1982; Aczel, 2019). N cycling is driven by a diverse of microbes (Simon and Klotz, 2013). Microbial diversity loss can affect N cycling process (Patkowska and Konopiński, 2013; Philippot et al., 2013; Li Z. et al., 2019). The previous studies indicated that abundance of microbes or functional genes associated with N cycling can be significantly changed by long-term agricultural management practices, which may indicate shifting of microbial function in agroecosystems (Doran et al., 1998; Sun et al., 2015). Therefore, studying the diversity of soil bacteria and changes of N cycling functional taxa is important for estimation of microbial function and prediction of soil function after a long-term agriculture management. The goal of the study was to evaluate the variation of bacterial community composition and N cycling bacterial taxa in different agricultural management practices under long-term (40 years) conservation management and temporal patterns during the cotton production cycle in West Tennessee. We hypothesized that (1) bacterial diversity would be greater following long-term (e.g., decades) of cover cropping and fertilization practices, whereas conventional (i.e., intensive) tillage would decrease bacterial diversity; (2) the bacterial community would be structured accordingly under long-term soil management practices but vary with time throughout the cotton production cycle; and (3) conventional tillage would decrease the relative abundances of denitrifying bacteria, and inorganic N fertilization will decrease relative abundance of N-fixing bacteria but increasing the nitrifying bacteria and denitrifying bacteria. Cover cropping would increase all the relative abundances of functional bacteria taxa. We used 16S rRNA high-throughput amplicon sequencing to estimate bacterial diversity and relative abundance of various taxonomic groups and structural equation modeling to associate these distribution patterns with management practice and potential roles of bacteria functional taxa in N cycling.

### Site Description and Sample Collection

The ongoing long-term conservation management experimental site was established in 1981 and is a randomized complete block (RCBD) with split-split plot design located in Jackson, TN, United States (35°37′23.1″N 88°50′47.4″W) (Li et al., 2021). The soil was Lexington silt loam (fine-silty, mixed, thermic, Ultic Hapludalfs). Inorganic nitrogen fertilizer (ammonium nitrate, NH_4_NO_3_) has been applied at two levels of nitrogen (NH_4_NO_3_ at 0 and 67 kg ha^–1^ or 60 lb acre^–1^) based on weight of N as the main plots and were divided into three subplots that contained three kinds of cover crop treatments (hairy vetch, *Vicia villosa* Roth; winter wheat, *Triticum aestivum* L.; and no cover). Each subplot contained two tillage treatments (conventional tillage and no tillage). The tillage was performed before planting cotton using a standard disk harrow followed by smoothing and breaking up of clods by a triple-K harrow. There were four replications in each of 12 treatments (Supplementary Figure 1). The collected samples were coded with the following form: “sampling month,” “type of cover crop,” “tillage or not,” “fertilization or not,” and “serial number of replicates,” e.g., M_NCCN0_1 represents May, no cover, conventional tillage with no fertilization treatment of replicate plot 1, which was sampled in May; O_VNTN60_4 is sampled in October, vetch-covered, no tillage with fertilization with 67 kg ha^–1^, replicate plot 4; and D_WCN0_3 is sampled in December, wheat-covered, conventional tillage without fertilization, replicate plot 3 (see Supplementary Table 4 for details).

The field sampling occurred four times during the cotton production cycle in 2019. The first sampling was made on May 21, 2019, shortly after burn-down of the cover crop and just before planting cotton and conventional tillage in spring. The second sampling was on June 12, 2019, after planting cotton and tillage but before fertilization in mid-summer. The third collection occurred on October 9, 2019, at the peak of cotton growth in fall. The final sample collection was performed at the beginning of December just after harvest but before cover crop planting for next year. Each sample was processed for 16S rRNA gene amplicon sequencing.

### Pre-processing and Measurement of Soil Properties

Soil samples were taken from a depth of 0 to 10 cm using a 2.5-cm-diameter soil probe. About 10–15 samples were randomly taken within each plot, approximately 10–15 cm away from the crop row center. Each soil sample was mixed and passed through a 2-mm sieve to remove fine rocks, roots, and other debris. We used the 75% ethanol (v/v) to clean the sampling probes, gloves, and sieves every time we sample different plots and sieve different samples.

Soil pH was measured using a pH electrode at a 1:2 ratio of soil to water (Ultrabasic, Denver Instrument, Bohemia, NY, United States). Gravimetric water content (GWC; g^–1^ dry soil) was determined as mass loss of field moist soil after oven drying at 105°C for 48 h. Salt extracts were conducted with a potassium persulfate reagent overnight (80°C) (Doyle et al., 2004). Then, salt extracts are carried through two colorimetric assays to measure extractable nitrate (NO_3_^–^) and ammonium (NH_4_^+^) concentrations. Nitrate was determined using a Vanadium (III) chloride reagent (Doane and Horwáth, 2003), and ammonium was quantified using the Berthelot reaction (Rhine et al., 1998). Air temperature data were obtained from Jackson Experiment Station *via* National Weather Service Forecast Office, National Oceanic and Atmospheric Administration^1^.

### DNA Extraction

Total soil DNA was extracted using a DNeasy^®^ PowerLyzer^®^ PowerSoil^®^ kit (REF 12855, QIAGEN) from 0.25 g of soil per sample following the manufacturer’s instructions. After DNA extraction from 192 samples (including four replicates from four blocks), the DNA was dissolved in sterile DNA-free PCR grade water. DNA quality and concentration were measured using NanoDrop (ND-3300 Fluorospectrometer 83060-50, Thermo Fisher Scientific).

### Library Preparation and Illumina MiSeq Sequencing

PCR amplification was carried out at UT Genomic Center of The University of Tennessee, targeting V3–V4 of bacterial 16S rRNA gene with the index and adaptors. The primers were 341F (5′- CCTACGGGNGGCWGCAG-3′) and 785R (5′-GACTACHVGGGTATCTAATCC-3′). The protocol included the primer pair sequences for the V3 and V4 region that create a single amplicon of approximately 464 bp (Klindworth et al., 2013). The PCR reaction (25 μl per sample) was performed using Platinum Green Hot start master mix (12.5 μl of 2 × concentration) (Invitrogen™, Catalog No. 13001012, United States), 2.5 μl of microbial genomic DNA (5 ng/μl in 10 mM Tris, pH 8.5), and 5 μl each of forward and reverse primer. PCR conditions consisted of a pre-denaturation at 95°C for 5 min, followed by 25 cycles of denaturation at 95°C for 40 s, annealing at 55°C for 2 min, elongation at 72°C for 1 min, and held at 4°C until storage in the freezer (−20°C) for future use. The 2 × 300 reads of each sample were generated by an Illumina MiSeq platform performed at DNA Genomics Core, University of Tennessee (Bartram et al., 2011). MiSeq Control Software version 2.6.2.1 was used for initial data processing.

### Sequence Data Analyses

Illumina sequencing data were examined by FastQC to perform the quality control (Andrews, 2010). Raw reads were processed using the MOTHUR pipeline (v. 1.35.1) (Schloss Laboratory; University of Michigan, Ann Arbor, MI, United States). The analysis followed MiSeq SOP^2^ (Kozich et al., 2013). The step of *make.contigs* was used to assemble reads into contigs. High-quality contigs were aligned with SILVA bacterial reference database (v.132) using kmer searching. After removing chimeric sequences using the VSEARCH program (Rognes et al., 2016), sequences were classified using the Ribosomal Database Project Naive Bayesian classifier to reference sequences with a bootstrap value cutoff 80% (Wang et al., 2007). Sequences identified as Chloroplast, Mitochondria, unknown, Archaea, or Eukaryota were removed. Then, sequences with no more than 3% dissimilarity were clustered into one Operational Taxonomic Unit (OTU). The *make.shared* command was performed to generate taxonomic and OTU tables for further analysis (Schloss et al., 2009; Schloss, 2020). The sequences for each replicate were rarefied to 14,145 sequences to avoid the influence of sample size on diversity estimation. Results were analyzed using *phyloseq* (v1.30.0) and *vegan* (v 2.5.7) packages for diversity analyses and visualized in RStudio 1.1.463 interface.

### Alpha- and Beta-Diversity Measurement

Shannon–Wiener, the number of OTUs, and Pielou’s evenness diversity indices indicate species richness and evenness (Kim et al., 2017). The canonical analysis of principal coordinates analysis (CAP) was conducted to examine bacterial community structure between individual samples. Similarity percentage analysis (SIMPER) was also utilized to test percentage of dissimilarities for each species contribution at phylum level between two treatments (Grange and Smith, 2013).

### Structural Equation Modeling

Structural equation modeling (AMOS 27; IBM Corporation, Meadville, PA, United States) was conducted to test the relationships among environmental factors, bacteria abundance, and the agricultural managements. Path coefficients were tested by maximum likelihood estimation at *p* ≤ 0.05. Multivariate normality was evaluated by Kurtosis value ≤7. Model fit was evaluated by (1) the minimum discrepancy divided by its degrees of freedom in the range of 1–3 (Carmines and McIver, 1983), (2) the goodness of fit index close to 1 (Tanaka and Huba, 1985), (3) the comparative fit index close to 1 (Bentler, 1990), and (4) the root mean square error of approximation less than 0.05 (Browne and Cudeck, 1993). We followed the procedures of developing and modifying a structural equation model in Byrne (2013a) and Li L. et al. (2019). Briefly, we proposed an *a priori* model based on our hypotheses, tested if the existing pathways were significant and if necessary pathways were left out, and then adjusted the *a priori* model by dropping insignificant pathways and adding missing pathways taking into consideration of model fit and scientific rationality.

We used SEM in this study for several reasons. (1) SEM is a confirmatory method that can test the validity of an existing theoretical framework built upon background knowledge and previous studies (Byrne, 2013b). In our hypothesized model, we assumed that field treatments would have effects on soil properties (Li L. et al., 2019; Mueller and Hancock, 2019), and the soil properties would affect microbial abundance and diversity (Byrne, 2013b; Mueller and Hancock, 2019). (2) SEM can evaluate the complex relationships among multiple variables beyond the traditional multiple regression (Colman and Schimel, 2013). (3) SEM can quantify these causal relationships by generating standardized coefficients (Grace and Bollen, 2005).

### Statistical Analysis

The mixed model ANOVA was used to test the variations of diversity indices (Chao1, the number of OTUs, Shannon, and Pielou) across treatments. The 17 samples, namely, M_VCN0_1, M_VCN0_2, M_VCN0_3, M_VNTN60_4, J_VCN0_1, J_VCN0_2, J_VCN0_3, J_VNTN60_4, O_VCN0_1, O_VCN0_2, O_VCN0_3, O_VNTN60_4, D_VCN0_1, D_VCN0_2, D_VCN0_3, D_NCCN0_4, and D_VNTN60_4, were removed due to low quality scores (less than 20) poor assembly and alignment to the 16S rRNA reference sequences. The M_VCN0_4, J_VCN0_4, O_VCN0_4, and D_VCN0_4 within the treatment of vetch-covered, conventional tillage with no fertilization (VCN0) were also removed because that one sample cannot run statistical analysis. All these 21 removed samples were treated as missing value during following statistical analysis. After the step of quality control, a total of 171 of 192 samples were remained for the downstream analyses. The statistical analysis was conducted in SAS software with Glimmix procedure (SAS Institute Inc., Cary, NC, United States), and the normality, equal variance, and outliers were checked using UNIVARIATE procedure. The least square means compared with Fisher’s Least Significant Difference at 5% significance level and Tukey’s Honest Significant Difference (Tukey’s HSD) tests were performed to compare differences between each treatment. The tillage, fertilization, cover cropping, and their interaction effects were tested in this study (Supplementary Figure 2). The statistical test on seasonal variation with regard to seasonal sampling as repeated measures was based on randomized complete block (RCBD) split-split plot design. Furthermore, three-way permutational multivariate analysis of variance tests with 9,999 permutations were also conducted within each sampling dates in R, respectively, to estimate if a given treatment signal was significant or not. The insignificant terms, including three-way and two-way interactions, cover crop treatment across June, October, and December, were removed from the statistical model to increase the number of degrees of freedom due to no significant effect (*p* < 0.05). Only significant environmental factors (*p* < 0.05) were kept in the model. We use adjusted R-square to avoid overexplaining the variation in the model (Peres-Neto et al., 2006).

Spearman’s correlation was performed among bacteria at genus level known to be associated with N fixation, nitrification, and denitrification in each sampling date, and significant correlations (*p* < 0.05) were corrected using Benjamini-Hochberg (BH) method (Benjamini, 1995). All functional taxa mentioned in this study were found by genus in our samples as reported in previous studies (Pagan et al., 1975; Pichinoty et al., 1976; Carlson and Ingraham, 1983; Davey and Marchant, 1983; Awonaike et al., 1990; Gee et al., 1990; Urakami et al., 1995; Baumann et al., 1996; Albrecht et al., 1997; Schramm et al., 1999; Hurek and Reinhold-Hurek, 2003; Verbaendert et al., 2011; Kostka et al., 2012; Cua and Stein, 2014; Sun et al., 2016; Fukami et al., 2018; Cardoso and Kana, 2019; Liu et al., 2019; Zhang H. et al., 2019; Spieck et al., 2020).

## Results

### The Contribution of Bacterial Phyla to the Total Variations of Bacterial Composition Across Treatments

In total, 4,854,968; 5,495,390; 3,983,387; and 3,809,793 raw sequence reads were obtained from samples collected in May, June, October, and December 2019, respectively. A total of 38,038 OTUs with 97% identity were generated from 171 samples after rarefaction. The number of OTUs per sample ranged from 4,134 (D_VCN60_1) to 2,758 (O_NCNTN0_4).

**FIGURE 1:**
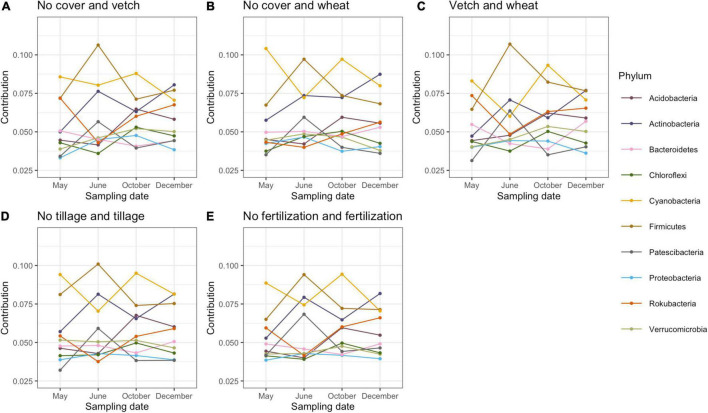
The contribution of bacteria phyla to the total dissimilarity of pairwise comparison among cover cropping **(A–C)**, tillage **(D)**, fertilization **(E)**, and across four seasons based on SIMPER (Analysis of Similarity) analysis.

*Proteobacteria*, *Acidobacteria*, *Actinobacteria*, *Verrucomicrobia*, and *Chloroflexi* were the most abundant bacterial phyla in this study (Supplementary Figure 2). SIMPER analysis (analysis of similarity of percentage) was used to estimate the contribution of each bacterial phylum and calculate the contribution of each bacterial taxonomic group to the dissimilarity of the whole bacterial community between different treatments (Warton et al., 2012). Contributions of more than 2% of the total variation of dominant phyla were included (Figure 1). All phyla showed a similar variation of contribution in five pair-wise comparisons (no cover and vetch, no cover and wheat, vetch and wheat, tillage and no tillage, and fertilization and no fertilization) across sampling dates. The contribution of *Firmicutes*, *Actinobacteria*, and *Patescibacteria* increased after planting cotton but, before fertilization, decreased at the peak of cotton growth and increased again after harvest. However, the *Cyanobacteria*, *Chloroflexi*, and *Acidobacteria* exhibited the opposite trend (Figure 1).

**FIGURE 2:**
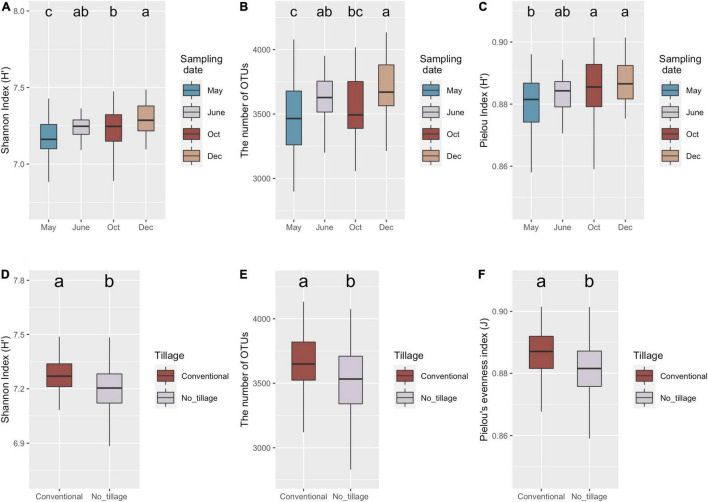
Variations of soil bacteria diversity under long-term management practices during the cotton production cycle. Boxplot of bacterial richness and evenness across four sampling dates **(A–C)** and tillage management. Shannon **(A,D)**, the number of OTUs **(B,E)**, and Pielou’s evenness **(C,F)** indices represented bacteria alpha-diversity, richness, and evenness, respectively. Minimum value, first quartile (Q1), median, third quartile (Q3), and maximum value were shown in the plot. The significance (*p* < 0.05) was represented by lower letters above the boxes; the boxes that shared same letter have no significant differences from each other.

The diversity, richness, and evenness of the bacterial communities in different samples were estimated by Shannon index (H), the number of OTUs, and Pielou’s index, respectively (Figure 2). No significant interaction effect was observed among tillage, fertilization, cover cropping, and sampling date (Supplementary Table 1). Only sampling date and tillage management had significant influence on bacterial richness and evenness (Supplementary Table 1). Shannon index (*p* < 0.001), number of OTUs (*p* < 0.01), and Pielou’s evenness (*p* < 0.01) varied across sampling dates (Figures 1A–C). Lowest alpha diversity was found after burn-down of cover crops (May) compared to samples collected after tillage but before fertilization (June) (i.e., richness, *p* < 0.05), after fertilization and at peak of cotton growth (October) (i.e., richness and evenness, *p* < 0.05), and after cotton harvest but before planting of the cover crops (December) (i.e., richness and evenness, *p* < 0.05). Soils sampled collected after cotton harvest had greater diversity relative to those at the peak of cotton growth because of a higher richness (*p* < 0.05). However, bacterial diversity showed no difference before (June) and after (October) in inorganic N fertilization plots. In addition, bacterial Shannon index (*p* < 0.01), numbers of OTUs (*p* < 0.01), and Pielou’s evenness (*p* < 0.05) were higher in conventional tillage compared to no tillage (Figures 2D,E). The results indicated that conventional tillage practices largely contributed to the change of bacterial alpha diversity over entire production cycle.

### Comparisons of Bacterial Community Structure Across Long-Term Tillage, Inorganic N Fertilization, and Cover Cropping Practices

**FIGURE 3:**
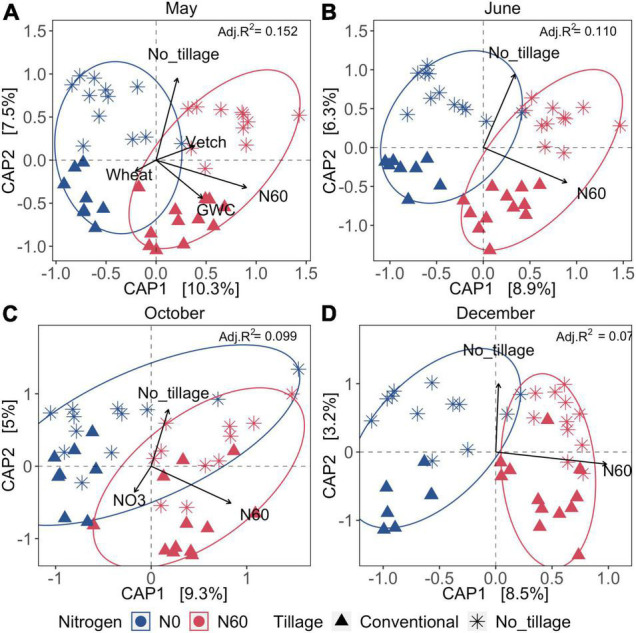
Canonical analysis of principal coordinates (CAP) across four seasons under tillage, fertilization, and cover cropping treatment. **(A–D)** The samples collected in May, June, October, and December, respectively. Colors represent the fertilization (0 or 67 kg ha^–1^ or 60 lb acre^–1^), and shapes represent the tillage treatment (no-till or intensive tillage). The significant environmental indicators are shown as arrows (*p* < 0.05). The Bray–Curtis dissimilarities were calculated to estimate the differences among samples. GWC was the gravimetric water content. NO3 was nitrate-N in ppm. N60 represented inorganic N fertilization. Vetch and wheat were the two cover cropping types. Adjusted *R*-square values indicate the total percentage explanation of variation by significant factors (shown as arrows) at each sampling date, which were shown on the right corner of each plot.

Overall, spatial variation of bacterial communities was significantly influenced by inorganic N fertilization and tillage across four sampling dates. Cover cropping, tillage, inorganic N fertilization, and soil water content explained 15.2% of dissimilarity of bacterial communities of soil samples collected after burn-down of cover crops (Samples collected in May) (Figure 3A). Bacterial communities were significantly influenced by the nitrate-N concentrations of the soil samples collected after inorganic N fertilization and at the peak of cotton growth (Samples collected in October) (Figure 3C). Approximately 7.1–15.2% of the variation of bacterial communities was explained by the environmental variables within the two CAP axis across May, June, October, and December (Figure 3 and Supplementary Table 2). The results suggested that the fertilization and tillage have a long-lasting impact on soil bacterial communities, and cover cropping only exerted the short-term effect after burn-down of cover crops in the aspect of one production cycles.

### Changes of Bacterial Genera Involved in N Fixation, Nitrification, and Denitrification Across Long-Term Management and Annual Environmental Change

**FIGURE 4:**
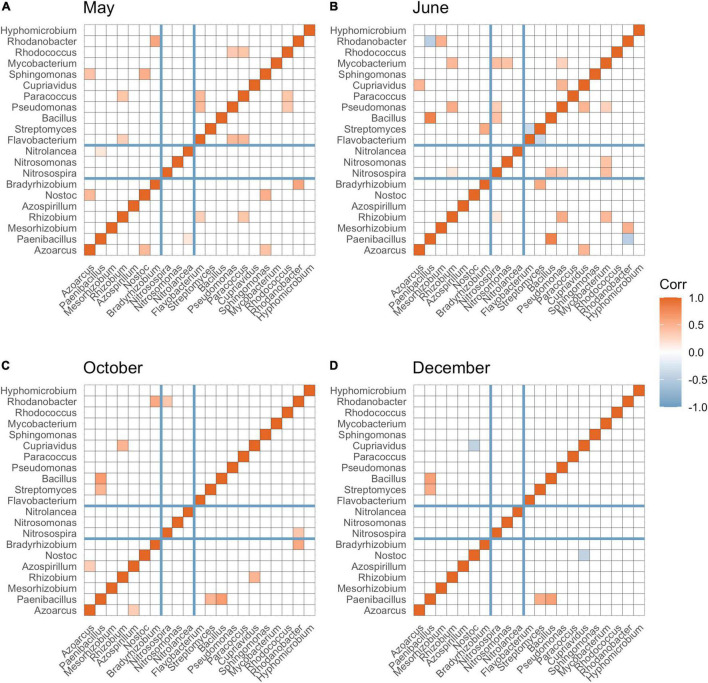
Spearman’s correlation among bacterial taxa at the genus level involved in N-fixation (*Azoarcus* to *Bradyrhizobium*), nitrification (*Nitrosospira* to *Nitrolancea*), and denitrification (*Flavobacterium* to *Hyphomicrobium*) across May **(A)**, June **(B)**, October **(C)**, and December **(D)**. Significant correlations (*p*-adjusted < 0.05) were colored by red (positive correlation) and blue (negative correlation). The darker the color, the greater absolute value of correlation coefficient. False discovery rate was controlled using Benjamini (1995) method.

N-fixing bacteria (including *Azoarcus*, *Paenibacillus*, *Rhizobium*, *Azospirillum*, *Nostoc*, and *Bradyrhizobium*), nitrifying bacteria (including *Nitrosospira*, *Nitrosomonas*, and *Nitrolancea*), and bacteria associated with denitrification (*Flavobacterium*, *Streptomyces*, *Bacillus*, *Pseudomonas*, *Paracoccus*, *Cupriavidus*, *Sphingomonas*, *Mycobacterium*, *Rhodococcus*, *Rhodanobacter*, and *Hyphomicrobium*) were detected in our soil samples. Overall, the greatest partition of significant connections was found between N-fixing bacteria and nitrifying bacteria or denitrifying bacteria compared to partition of connections between nitrifying and denitrifying bacteria. After burn-down of cover crops, approximately 9.10% of total significant correlations (*p* < 0.05) among all N groups were between N-fixing bacteria and nitrifying bacteria, and 45.45% numbers of total significant connections (*p* < 0.05) were between N-fixing bacteria and denitrifying bacteria (Figure 4A). After tillage but before fertilization, approximately 6.67 and 46.67% significant connections (*p* < 0.05) were observed among N-fixing bacteria and nitrifying bacteria, and N-fixing bacteria and denitrifying bacteria, respectively (Figure 4B). After fertilization and cotton harvest, most of the positive connections were observed between N fixing and denitrifying bacteria (Figures 4C,D).

**FIGURE 5:**
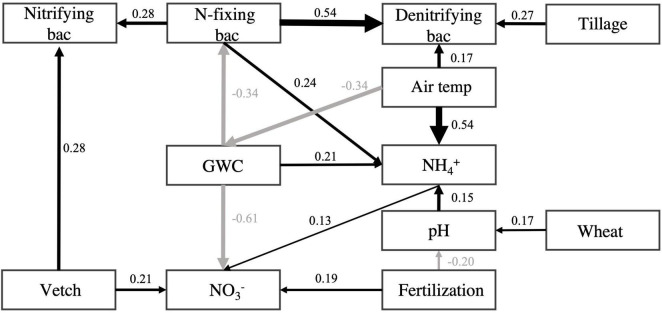
Structural equation model for N-fixing, nitrifying, and denitrifying bacteria. Boxes represent variables. Single-headed arrows represent causal relationships. Black arrows indicate positive effects, and gray arrows indicate negative effects. No arrows suggest no significant relationships between variables. All presented relationships are significant at *p* < 0.05. Numbers beside each arrow are standardized path coefficients (i.e., effect sizes). See Supplementary Table 3 for standardized total effects. The “Vetch” and “Wheat,” “Tillage,” and “Fertilization” were presenting the cover cropping, conventional tillage, and inorganic N fertilization treatment, respectively. Soil pH (pH), gravimetric water content (GWC), ammonia-N (NH_4_^+^), and nitrate-N (NO_3_^–^), air temperature (month average ahead of sampling date) presented in rectangular box.

As shown in the structural equation model for evaluating the influence of tillage, fertilization, and cover cropping on the relative abundance of N-fixing, nitrifying, and denitrifying bacteria, the abundances of N-fixing bacteria had positive effect on nitrifying (standardized path coefficient = 0.28, *p* < 0.001) and denitrifying bacteria (standardized path coefficient = 0.54, *p* < 0.001), respectively. As for the effect of cover crop, vetch-covered soil increased the abundance of nitrifying bacteria (standardized path coefficient = 0.28, *p* < 0.001). Tillage increased the abundances of denitrifying bacteria (standardized path coefficient = 0.27, *p* < 0.001). N-fixing bacteria were negatively influenced by soil moisture content (standardized total effect = −0.34, *p* < 0.001) and positively influenced by air temperature (standardized total effect = 0.11, *p* < 0.001) (Figure 5 and Supplementary Table 3).

## Discussion

### The Variation of Bacterial Diversity, Composition, and Structure Under Long-Term Management Practices

The contributions of bacteria phyla on variations among sampling dates were consistent across treatments, indicating that the communities are likely dominated by taxa that have acclimated to the ambient environment conditions and/or formed a dynamic steady pattern after the long-term soil management. *Actinobacteria* and *Firmicutes* were reported as ecologically relevant copiotroph-associated phyla (Fierer et al., 2012; Ramirez et al., 2012; Bastida et al., 2015), which may response to more organic matter input after burn-down of cover crops. *Cyanobacteria*, *Chloroflexi*, and *Acidobacteria* were as oligotrophic-associated phyla (Fierer et al., 2012; Ramirez et al., 2012; Pepe-Ranney et al., 2016), which may be disturbed when soil with limited nutrient status, such as after peak of cotton growth.

Previous research has indicated that no tillage can increase soil microbial diversities, which benefit from greater soil moisture and lower fluctuation of soil temperature created by no tillage (Wang et al., 2016). No tillage also can exhibit no effect on bacteria diversity in other studies (Li et al., 2018). Here, bacterial alpha diversity in our study increased under conventional tillage. The possible reason might be that conventional tillage management is known to disturb the stability of soil aggregates and release physically protected organic matter utilized by microbes (Rong et al., 2017; Bu et al., 2020).

In addition, previous studies show that tillage can improve soil aeration, total porosity, and oxygen diffusion rate, providing a suitable environment for aerobic microbial growth and accelerated decomposition of soil organic matter as substrate and energy source for soil microbes (Linn and Doran, 1984; Khan, 1996; Simmons and Coleman, 2008). In addition, different microbial functional groups harbored at different soil depths and tillage practices might relocate microbial groups in deeper soil to the surface soil, leading to increase in bacterial richness and evenness of bacterial communities.

No significant effect of cover cropping and inorganic N fertilization on bacterial alpha can be observed, which contradicts the common assertion that cover cropping and inorganic N fertilization can increase the diverse of organic matter input, prevent nutrient leaching, and keep the soil physical structure, which would provide a positive effect on for bacteria growth and diversity (Novara et al., 2020). Incorporated organic matter may result in only a short-term change on bacteria diversity through favor relatively corticotropic taxa contribute to largest partition communities. The N input can decrease the bacterial OTU richness due to the soil acidification (Zeng et al., 2016; Dai et al., 2018). The fact that there are no changes in bacterial richness and evenness under inorganic N fertilization is consistent with the result that the no obvious decrease on soil pH in our long-term managed field.

Meanwhile, conventional tillage and fertilization exerted a lasting effect on bacterial structure throughout the growing cycle. The observed lasting effect of field management practices over decades was most pronounced in tillage and/or fertilization and was consistent with previous studies (Marschner et al., 2003; Toda and Uchida, 2017). In addition, nitrate-N input significantly explained variation of bacterial communities among samples after fertilization (October sampling date). In previous studies, microbial biomass, bacterial community structure, and specific taxa can respond to inorganic fertilization management (Ramirez et al., 2010; Geisseler and Scow, 2014; Zhou et al., 2017). For example, increased nitrogen availability would likely suppress N-fixing bacteria but stimulate nitrifier and denitrifier populations and then shift the structure of bacteria community (Ouyang et al., 2018; Norton and Ouyang, 2019).

The effect of cover cropping on bacterial community structure was observed only after the burn-down of cover crops which exhibited a relatively short-term effect (approximately 1 month) compared to inorganic N fertilization and conventional tillage. At this site, cover crops were planted and grown in winter and spring seasons as one of several experimental treatments and the cover crops residues were left on the soil surface. Winter wheat possessed a high C:N ratio compared to hairy vetch biomass, which likely affected the quality and quantity of carbon, nitrogen, and other nutrient resources available for bacteria (Vukicevich et al., 2016; Schmidt et al., 2018). The plant residues with different nutrient compositions could have a large impact in reshaping the soil bacterial community structure (Marschner et al., 2003). For example, the soil enriched with recalcitrant substrates often exhibits enrichment in microbes that have the ability to secrete extracellular enzymes to degrade complex polymers (e.g., cellulase) (Marschner et al., 2003). Meanwhile, this relatively short-term effect from cover cropping on reshaping soil bacterial communities structure might relate to increasing of soil moisture content (Acharya et al., 2019).

### The Response of Bacterial Taxa Involved in N Cycling to Management Practices

A conceptual model, “N saturation hypothesis,” states that an N supply that exceeds biological demand could result in the loss of N from the ecosystem (Aber et al., 1998; Galloway et al., 2003; Pajares and Bohannan, 2016). N addition or loss can potentially be affected by abundances of functional taxa. Here, we observed N-fixing taxa had a substantial positive effect on the relative abundance of denitrifying bacterial groups across the four sampling times. These results may indicate that positive correlation between the relative abundances N fixer and denitrifiers, and N fixer and nitrifiers might be the reason to provide N cycled by microbes and meet the N needs of soil bacteria.

Nitrification process driven by nitrifiers apparently responded to crop residues with low C:N ratio input. Low C:N ratio of vetch compared to that of other cover crops like wheat provided more readily utilizable substrate for bacterial decomposition. N input by N-fixing cover crops can also likely increased the activity and abundance of nitrifiers that use reduced N forms as an oxidizable energy source and, subsequently, by heterotrophs that utilize the oxidized nitrogen as an alternate terminal electron acceptor (Songjuan et al., 2021). Microbial N transformation accelerated by vetch cover crop was found in the same study site (Li et al., 2021). Our result is also consistent with the finding that functional activity (transcript copy abundances) of bacterial N cycling groups, such as *amoA* genes involved in nitrification process, was promoted by hairy vetch plantation (Hu et al., 2021a).

In previous research, conventional tillage disrupted the soil aggregate structure, resulting in increase in the abundance of denitrifiers (Dick, 1992; Buckley and Schmidt, 2001; Kladivko, 2001; Lienhard et al., 2013). In this study, conventional tillage showed a positive effect on denitrifier abundance, in agreement with previous studies that observed greater denitrification gene abundance under the conditions similar to this study (Wang et al., 2019). This may be due to denitrifiers brought from deeper soil being brought to the surface soil by conventional tillage (Kladivko, 2001; Holland, 2004; Wang et al., 2017).

According to the SEM results, soil moisture content can negatively influence the abundance of N-fixing bacteria. Although nitrogenase mediates, N fixation process is oxygen-sensitive (Gallon, 1981). The process can be conducted by aerobic N-fixing microbes (Barney, 2020). For example, *Azospirillum* found in our study was reported as aerobic nitrogen-fixing bacteria (Huergo et al., 2008), which may benefit from low soil moisture. The free-living N-fixing bacteria (e.g., *Nostoc*) can survive from drought stress (Fuhrmann et al., 1986). In addition, previous studies show that the survival and distribution of N-fixing bacteria were influenced not necessarily restricted by anaerobic conditions due to higher soil moisture; it also needs to consider the initial moisture content and moisture stress in water-filled pores of soil microhabitat (Postma et al., 1989; Zahran, 1999).

## Conclusion

Soil bacterial communities were influenced by long-term agricultural management practices and seasonal climate variation based on 1-year field investigation at The University of Tennessee West Research and Education Center. Long-term conventional tillage, inorganic N fertilization, and cover cropping not only did not dampen the alpha diversity of bacteria communities but also can reshape bacteria community structure and exert different lasting effect (relatively short-term from cover cropping and long-term from fertilization and tillage). Meanwhile, oligotroph-associated bacteria and copiotroph-associated bacteria phyla show a steady dynamic pattern, indicating that acclimation of bacterial community may occur in a long-term managed soil ecosystem. In addition, the management practices may indirectly enhance the correlations between N cycling functional groups. The results of the correlation between bacterial N cycling groups disclose that bacteria community built the network for providing N and meet microbial N needs in the long term. In this study, variation of relative abundances of N cycling–related functional taxa indirectly reflects the roles of bacteria, which is the limitation of this study. Direct evidence (e.g., transcriptome) is needed to get insight into bacterial functional activities for future studies.

## Data Availability Statement

The datasets presented in this study can be found in online repositories. The names of the repository/repositories and accession number(s) can be found in the article/Supplementary Material.

## Author Contributions

ND, JZ, MR, and SS designed the experiment. ND, XL, and AF conducted the experiment. ND and LL analyzed the data. ND, LL, JZ, MR, and SS revised the manuscript. All authors contributed to the article and approved the submitted version.

## Conflict of Interest

The authors declare that the research was conducted in the absence of any commercial or financial relationships that could be construed as a potential conflict of interest.

## Publisher’s Note

All claims expressed in this article are solely those of the authors and do not necessarily represent those of their affiliated organizations, or those of the publisher, the editors and the reviewers. Any product that may be evaluated in this article, or claim that may be made by its manufacturer, is not guaranteed or endorsed by the publisher.

## Footnotes

1
https://www.weather.gov/

2
https://mothur.org/wiki/miseq_sop/

## Supplementary Material

Supplementary Figure 1 Field design (RCBD with split-split plot). The plots are 40′ × 26.6′ with 20′ alleys. Total average with alleys 4.5 ac, main plot: nitrogen fertilization rate (0, 60 lb N/ac), Subplot: cover crop, no-cover, hairy vetch, crimson clover, wheat. Sub-sub plot: Tillage; C, conventional till; NT, no-till.

Supplementary Figure 2 The relative abundance of bacterial phyla, including *Proteobacteria* **(A)**, *Acidobacteria* **(B)**, *Actinobacteria* **(C)**, *Verrucomicrobia* **(D)**, *Choroflexi* **(E)**, *Planctomycetes* **(F)**, *Bacteroidetes* **(G)**, *Firmicutes* **(H)**, *Gemmatimonadetes* **(I)**, *Patescibacteria* **(J)**, *Cynaobacteria* **(K)**, *Nitrospirae* **(L)** across treatments collected in May, June, October, and December.

Supplementary Table 1 Statistical results of diversity indices across sampling dates, tillage, N fertilization, and cover cropping.

Supplementary Table 2 PERMANOVA results for the significant factors influencing bacterial community structure across each sampling date.

Supplementary Table 3 Standard total effect for SEM.

Supplementary Table 4 Coding for sample ID.

Supplementary Table 5 The data for SEM.

## References

[B1] Aber J. McDowell W. Nadelhoffer K. Magill A. Berntson G. Kamakea M. (1998). Nitrogen saturation in temperate forest ecosystems: hypotheses revisited. *Bioscience* 48 921–934. 10.2307/1313296

[B2] Acharya B. S. Dodla S. Gaston L. A. Darapuneni M. Wang J. J. Sepat S. (2019). Winter cover crops effect on soil moisture and soybean growth and yield under different tillage systems. *Soil Tillage Res.* 195:104430. 10.1016/j.still.2019.104430

[B3] Aczel M. R. (2019). What is the nitrogen cycle and why is it key to life? *Front. Young Minds* 7:41. 10.3389/frym.2019.00041

[B4] Albrecht A. Ottow J. Benckiser G. Sich I. Russow R. (1997). Incomplete denitrification (NO and N2O) from nitrate by Streptomyces violaceoruber and S. nitrosporeus revealed by acetylene inhibition and 15N gas chromatography-quadrupole mass spectrometry analyses. *Naturwissenschaften* 84 145–147. 10.1007/s001140050365

[B5] Anand M. Baidyanath K. Dina N. (2015). Cyanobacterial consortium in the improvement of maize crop. *Int. J. Curr. Microbiol. Appl. Sci.* 4 264–274.

[B6] Andrews S. (2010). *FastQC: A Quality Control Tool for High Throughput Sequence Data.* Available online at: http://www.bioinformatics.babraham.ac.uk/projects/fastqc/

[B7] Awonaike K. Kumarasinghe K. Danso S. (1990). Nitrogen fixation and yield of cowpea (Vigna unguiculata) as influenced by cultivar and Bradyrhizobium strain. *Field Crops Res.* 24 163–171. 10.1016/0378-4290(90)90035-a

[B8] Bardgett R. D. Shine A. (1999). Linkages between plant litter diversity, soil microbial biomass and ecosystem function in temperate grasslands. *Soil Biol. Biochem.* 31 317–321. 10.1016/s0038-0717(98)00121-7

[B9] Barney B. M. (2020). Aerobic nitrogen-fixing bacteria for hydrogen and ammonium production: current state and perspectives. *Appl. Microbiol. Biotechnol.* 104 1383–1399. 10.1007/s00253-019-10210-9 31879824

[B10] Bartram A. K. Lynch M. D. Stearns J. C. Moreno-Hagelsieb G. Neufeld J. D. (2011). Generation of multimillion-sequence 16S rRNA gene libraries from complex microbial communities by assembling paired-end illumina reads. *Appl. Environ. Microbiol.* 77 3846–3852. 10.1128/AEM.02772-10 21460107 PMC3127616

[B11] Bastida F. Selevsek N. Torres I. F. Hernández T. García C. (2015). Soil restoration with organic amendments: linking cellular functionality and ecosystem processes. *Sci. Rep.* 5:15550. 10.1038/srep15550 26503516 PMC4621494

[B12] Basu S. Kumar G. Chhabra S. Prasad R. (2021). “Role of soil microbes in biogeochemical cycle for enhancing soil fertility,” in *New and Future Developments in Microbial Biotechnology and Bioengineering*, ed. Rodrigues A. (Amsterdam: Elsevier), 149–157. 10.1016/b978-0-444-64325-4.00013-4

[B13] Baumann B. Snozzi M. Zehnder A. Van Der Meer J. R. (1996). Dynamics of denitrification activity of *Paracoccus denitrificans* in continuous culture during aerobic-anaerobic changes. *J. Bacteriol.* 178 4367–4374. 10.1128/jb.178.15.4367-4374.1996 8755862 PMC178201

[B14] Bending G. D. Turner M. K. Jones J. E. (2002). Interactions between crop residue and soil organic matter quality and the functional diversity of soil microbial communities. *Soil Biol. Biochem.* 34 1073–1082. 10.1016/s0038-0717(02)00040-8

[B15] Benjamini Y. (1995). Benjamini and Hochberg methods. *J. R. Stat. Soc. Ser. B Methodol.* 57 289–300.

[B16] Bentler P. M. (1990). Comparative fit indexes in structural models. *Psychol. Bull.* 107 238–46. 10.1037/0033-2909.107.2.238 2320703

[B17] Brown M. E. Chang M. C. (2014). Exploring bacterial lignin degradation. *Curr. Opin. Chem. Biol.* 19 1–7. 10.1016/j.cbpa.2013.11.015 24780273

[B18] Browne M. W. Cudeck R. (1993). “Alternative ways of assessing model fit,” in *Testing Structural Equation Models*, eds Bollen K. A. Long J. S. (Beverly Hills: Sage), 111–135.

[B19] Bruns M. A. Stephen J. R. Kowalchuk G. A. Prosser J. I. Paul E. A. (1999). Comparative diversity of ammonia oxidizer 16S rRNA gene sequences in native, tilled, and successional soils. *Appl. Environ. Microbiol.* 65 2994–3000. 10.1128/AEM.65.7.2994-3000.1999 10388694 PMC91447

[B20] Bu R. Ren T. Lei M. Liu B. Li X. Cong R. (2020). Tillage and straw-returning practices effect on soil dissolved organic matter, aggregate fraction and bacteria community under rice-rice-rapeseed rotation system. *Agric. Ecosyst. Environ.* 287:106681. 10.1016/j.agee.2019.106681

[B21] Buckley D. Schmidt T. (2001). The structure of microbial communities in soil and the lasting impact of cultivation. *Microb. Ecol.* 42 11–21. 10.1007/s002480000108 12035077

[B22] Byrne B. M. (2013a). *Structural Equation Modeling with AMOS: Basic Concepts, Applications, and Programming*, 2nd Edn. New York: Routledge.

[B23] Byrne B. M. (2013b). *Structural Equation Modeling with Mplus: Basic Concepts, Applications, and Programming.* Miltion Park: Routledge.

[B24] Cardoso N. C. Kana B. D. (2019). The contribution of the NarB and NarGHI enzymes to nitrate reduction in Mycobacterium smegmatis. *Res. Sq.* [Preprint]. [Preprint]., 10.21203/rs.2.9260/v1

[B25] Carlson C. A. Ingraham J. L. (1983). Comparison of denitrification by *Pseudomonas stutzeri*, *Pseudomonas aeruginosa*, and *Paracoccus denitrificans*. *Appl. Environ. Microbiol.* 45 1247–1253. 10.1128/aem.45.4.1247-1253.1983 6407395 PMC242446

[B26] Carmines E. G. McIver J. P. (1983). An introduction to the analysis of models with unobserved variables. *Polit. Methodol.* 9 51–102.

[B27] Colman B. P. Schimel J. P. (2013). Drivers of microbial respiration and net N mineralization at the continental scale. *Soil Biol. Biochem.* 60 65–76. 10.1016/j.soilbio.2013.01.003

[B28] Cua L. S. Stein L. Y. (2014). Characterization of denitrifying activity by the alphaproteobacterium, *Sphingomonas wittichii* RW1. *Front. Microbiol.* 5:404. 10.3389/fmicb.2014.00404 25147547 PMC4123721

[B29] Dai Z. Su W. Chen H. Barberán A. Zhao H. Yu M. (2018). Long-term nitrogen fertilization decreases bacterial diversity and favors the growth of Actinobacteria and *Proteobacteria* in agro-ecosystems across the globe. *Glob. Chang. Biol.* 24 3452–3461. 10.1111/gcb.14163 29645398

[B30] Davey A. Marchant H. J. (1983). Seasonal variation in nitrogen fixation by Nostoc commune Vaucher at the Vestfold Hills, Antarctica. *Phycologia* 22 377–385. 10.2216/i0031-8884-22-4-377.1

[B31] Dick R. P. (1992). A review: long-term effects of agricultural systems on soil biochemical and microbial parameters. *Agric. Ecosyst. Environ.* 40 25–36. 10.3389/fmicb.2018.01929 30210462 PMC6119716

[B32] Doane T. A. Horwáth W. R. (2003). Spectrophotometric determination of nitrate with a single reagent. *Anal. Lett.* 36, 2713–2722. 10.1081/AL-120024647

[B33] Doran J. Elliott E. Paustian K. (1998). Soil microbial activity, nitrogen cycling, and long-term changes in organic carbon pools as related to fallow tillage management. *Soil Tillage Res.* 49 3–18. 10.1016/s0167-1987(98)00150-0

[B34] Doyle A. Weintraub M. N. Schimel J. P. (2004). Persulfate digestion and simultaneous colorimetric analysis of carbon and nitrogen in soil extracts. *Soil Sci. Soc. Am. J.* 68, 669–676. 10.2136/sssaj2004.6690

[B35] Etemadi F. Hashemi M. Zandvakili O. Dolatabadian A. Sadeghpour A. (2018). Nitrogen contribution from winter-killed faba bean cover crop to spring-sown sweet corn in conventional and no-till systems. *Agron. J.* 110 455–462. 10.2134/agronj2017.08.0501

[B36] Fierer N. Lauber C. L. Ramirez K. S. Zaneveld J. Bradford M. A. Knight R. (2012). Comparative metagenomic, phylogenetic and physiological analyses of soil microbial communities across nitrogen gradients. *ISME J.* 6 1007–1017. 22134642 10.1038/ismej.2011.159 PMC3329107

[B37] Finney D. M. Buyer J. S. Kaye J. P. (2017). Living cover crops have immediate impacts on soil microbial community structure and function. *J. Soil Water Conserv.* 72 361–373. 10.2489/jswc.72.4.361

[B38] Fuhrmann J. Davey C. Wollum A. (1986). Desiccation tolerance of clover rhizobia in sterile soils. *Soil Sci. Soc. Am. J.* 50 639–644. 10.2136/sssaj1986.03615995005000030019x

[B39] Fukami J. Cerezini P. Hungria M. (2018). Azospirillum: benefits that go far beyond biological nitrogen fixation. *AMB Express* 8:73. 10.1186/s13568-018-0608-1 29728787 PMC5935603

[B40] Gallon J. (1981). The oxygen sensitivity of nitrogenase: a problem for biochemists and micro-organisms. *Trends Biochem. Sci.* 6 19–23. 10.1016/0968-0004(81)90008-6

[B41] Galloway J. N. Aber J. D. Erisman J. W. Seitzinger S. P. Howarth R. W. Cowling E. B. (2003). The nitrogen cascade. *Bioscience* 53 341–356.

[B42] Gao Y. Du X. Xu W. Fan R. Zhang X. Yang S. (2020). Fungal diversity in deep sea sediments from East Yap Trench and their denitrification potential. *Geomicrobiol. J.* 37 848–858. 10.1080/01490451.2020.1789778

[B43] Gee C. S. Pfeffer J. T. Suidan M. T. (1990). Nitrosomonas and Nitrobacter interactions in biological nitrification. *J. Environ. Eng.* 116 4–17.

[B44] Geisseler D. Scow K. M. (2014). Long-term effects of mineral fertilizers on soil microorganisms–A review. *Soil Biol. Biochem.* 75 54–63. 10.1016/j.soilbio.2014.03.023

[B45] Grace J. B. Bollen K. A. (2005). Interpreting the results from multiple regression and structural equation models. *Bull. Ecol. Soc. Am.* 86 283–295. 10.1890/0012-9623(2005)86[283:itrfmr]2.0.co;2

[B46] Grange L. J. Smith C. R. (2013). Megafaunal communities in rapidly warming fjords along the West Antarctic Peninsula: hotspots of abundance and beta diversity. *PLoS One* 8:e77917. 10.1371/journal.pone.0077917 24312442 PMC3848936

[B47] Greenwood D. (1982). Nitrogen supply and crop yield: the global scene. *Plant Soil* 67 45–59. 10.1007/978-94-009-7639-9_4

[B48] Holland J. M. (2004). The environmental consequences of adopting conservation tillage in Europe: reviewing the evidence. *Agric. Ecosyst. Environ.* 103 1–25. 10.1016/j.agee.2003.12.018

[B49] Hu J. Jin V. L. Konkel J. Y. Schaeffer S. M. Schneider L. G. DeBruyn J. M. (2021a). Soil health management enhances microbial nitrogen cycling capacity and activity. *mSphere* 6 e01237–20. 10.1128/mSphere.01237-20 33441406 PMC7845608

[B50] Hu J. Richwine J. D. Keyser P. D. Li L. Yao F. Jagadamma S. (2021b). Nitrogen fertilization and native C4 grass species alter abundance, activity, and diversity of soil diazotrophic communities. *Front. Microbiol.* 12:675693. 10.3389/fmicb.2021.675693 34305840 PMC8297707

[B51] Huergo L. F. Monteiro R. A. Bonatto A. C. Rigo L. U. Steffens M. Cruz L. M. (2008). “Regulation of nitrogen fixation in *Azospirillum brasilense*,” in *Azospirillum sp.: Cell Physiology, Plant Interactions and Agronomic Research in Argentina*, eds Cassán F. D. Salamone G. (Buenos Aires: Asociación Argentina de Microbiologia), 17–35.

[B52] Hurek T. Reinhold-Hurek B. (2003). Azoarcus sp. strain BH72 as a model for nitrogen-fixing grass endophytes. *J. Biotechnol.* 106 169–178. 10.1016/j.jbiotec.2003.07.010 14651859

[B53] Jiménez-Bueno N. Valenzuela-Encinas C. Marsch R. Ortiz-Gutiérrez D. Verhulst N. Govaerts B. (2016). Bacterial indicator taxa in soils under different long-term agricultural management. *J. Appl. Microbiol.* 120 921–933. 10.1111/jam.13072 26808352

[B54] Khan A. (1996). Influence of tillage on soil aeration. *J. Agron. Crop Sci.* 177 253–259. 10.1111/j.1439-037x.1996.tb00243.x

[B55] Kibblewhite M. Ritz K. Swift M. J. (2008). Soil health in agricultural systems. *Philos. Trans. R. Soc. Lond. B Biol. Sci*. 363 685–701. 17785275 10.1098/rstb.2007.2178 PMC2610104

[B56] Kim B. R. Shin J. Guevarra R. B. Lee J. H. Kim D. W. Seol K. H. (2017). Deciphering diversity indices for a better understanding of microbial communities. *J. Microbiol. Biotechnol.* 27 2089–2093. 10.4014/jmb.1709.09027 29032640

[B57] Kladivko E. J. (2001). Tillage systems and soil ecology. *Soil Tillage Res.* 61 61–76. 10.1016/s0167-1987(01)00179-9

[B58] Klindworth A. Pruesse E. Schweer T. Peplies J. Quast C. Horn M. (2013). Evaluation of general 16S ribosomal RNA gene PCR primers for classical and next-generation sequencing-based diversity studies. *Nucleic Acids Res.* 41:e1. 10.1093/nar/gks808 22933715 PMC3592464

[B59] Kostka J. E. Green S. J. Rishishwar L. Prakash O. Katz L. S. Mariño-Ramírez L. (2012). Genome sequences for six Rhodanobacter strains, isolated from soils and the terrestrial subsurface, with variable denitrification capabilities. *J. Bacteriol.* 194 4461–4462. 10.1128/JB.00871-12 22843592 PMC3416251

[B60] Kozich J. J. Westcott S. L. Baxter N. T. Highlander S. K. Schloss P. D. (2013). Development of a dual-index sequencing strategy and curation pipeline for analyzing amplicon sequence data on the MiSeq Illumina sequencing platform. *Appl. Environ. Microbiol.* 79 5112–5120. 10.1128/AEM.01043-13 23793624 PMC3753973

[B61] Legrand F. Picot A. Cobo-Díaz J. F. Carof M. Chen W. Le Floch G. (2018). Effect of tillage and static abiotic soil properties on microbial diversity. *Appl. Soil Ecol.* 132 135–145. 10.1016/j.apsoil.2018.08.016

[B62] Li H. Zhang Y. Yang S. Wang Z. Feng X. Liu H. (2019). Variations in soil bacterial taxonomic profiles and putative functions in response to straw incorporation combined with N fertilization during the maize growing season. *Agric. Ecosyst. Environ.* 283:106578. 10.1016/j.agee.2019.106578

[B63] Li L. Konkel J. Jin V. L. Schaeffer S. M. (2021). Conservation management improves agroecosystem function and resilience of soil nitrogen cycling in response to seasonal changes in climate. *Sci. Total Environ.* 779:146457. 10.1016/j.scitotenv.2021.146457 34030284

[B64] Li L. Wilson C. B. He H. Zhang X. Zhou F. Schaeffer S. M. (2019). Physical, biochemical, and microbial controls on amino sugar accumulation in soils under long-term cover cropping and no-tillage farming. *Soil Biol. Biochem.* 135 369–378. 10.1016/j.soilbio.2019.05.017

[B65] Li L. Xi Y. Chen E. He L. Wang L. Xiao X. (2018). Effects of tillage and green manure crop on composition and diversity of soil microbial community. *J. Ecol. Rural Environ.* 34 342–348.

[B66] Li Z. Tian D. Wang B. Wang J. Wang S. Chen H. Y. (2019). Microbes drive global soil nitrogen mineralization and availability. *Glob. Chang. Biol.* 25 1078–1088. 10.1111/gcb.14557 30589163

[B67] Lienhard P. Tivet F. Chabanne A. Dequiedt S. Lelièvre M. Sayphoummie S. (2013). No-till and cover crops shift soil microbial abundance and diversity in Laos tropical grasslands. *Agron. Sustain. Dev.* 33 375–384. 10.1007/s13593-012-0099-4

[B68] Linn D. Doran J. (1984). Aerobic and anaerobic microbial populations in no-till and plowed soils. *Soil Sci. Soc. Am. J.* 48 794–799. 10.2136/sssaj1984.03615995004800040019x

[B69] Liu S. Coyne M. Grove J. Flythe M. (2018). Nitrogen, season, and tillage management influence ammonia oxidizing bacterial communities in long-term maize. *Appl. Soil Ecol.* 129 98–106. 10.1016/j.apsoil.2018.05.002

[B70] Liu X. Li Q. Li Y. Guan G. Chen S. (2019). Paenibacillus strains with nitrogen fixation and multiple beneficial properties for promoting plant growth. *PeerJ* 7:e7445. 10.7717/peerj.7445 31579563 PMC6761918

[B71] Liu X. Li Y. Ren X. Chen B. Zhang Y. Shen C. (2020). Long-term greenhouse cucumber production alters soil bacterial community structure. *J. Soil Sci. Plant Nutr.* 20 306–321. 10.1007/s42729-019-00109-9

[B72] Logsdon S. D. Karlen D. L. (2004). Bulk density as a soil quality indicator during conversion to no-tillage. *Soil Tillage Res.* 78 143–149. 10.1016/j.still.2004.02.003

[B73] Marschner P. Kandeler E. Marschner B. (2003). Structure and function of the soil microbial community in a long-term fertilizer experiment. *Soil Biol. Biochem.* 35 453–461. 10.1016/s0038-0717(02)00297-3

[B74] Mbuthia L. W. (2014). *Long-Term Impacts of Tillage, Cover Crops, and Nitrogen Rates on Microbial Community Dynamics and Soil Quality Parameters under Continuous Cotton Production in West Tennessee.* Ph.D. thesis. Knoxville: University of Tennessee.

[B75] Meliani A. Bensoltane A. Mederbel K. (2012). Microbial diversity and abundance in soil: related to plant and soil type. *Am. J. Plant Nutr. Fertil. Technol.* 2 10–18. 10.3923/ajpnft.2012.10.18

[B76] Mueller R. O. Hancock G. R. (2019). *Structural Equation Modeling.* Miltion Park: Routledge.

[B77] Navarro-Noya Y. E. Gómez-Acata S. Montoya-Ciriaco N. Rojas-Valdez A. Suárez-Arriaga M. C. Valenzuela-Encinas C. (2013). Relative impacts of tillage, residue management and crop-rotation on soil bacterial communities in a semi-arid agroecosystem. *Soil Biol. Biochem.* 65 86–95. 10.1016/j.soilbio.2013.05.009

[B78] Norton J. Ouyang Y. (2019). Controls and adaptive management of nitrification in agricultural soils. *Front. Microbiol.* 10:1931. 10.3389/fmicb.2019.01931 31543867 PMC6728921

[B79] Novara A. Catania V. Tolone M. Gristina L. Laudicina V. A. Quatrini P. (2020). Cover crop impact on soil organic carbon, nitrogen dynamics and microbial diversity in a Mediterranean semiarid vineyard. *Sustainability* 12:3256. 10.3390/su12083256

[B80] Ouyang Y. Evans S. E. Friesen M. L. Tiemann L. K. (2018). Effect of nitrogen fertilization on the abundance of nitrogen cycling genes in agricultural soils: a meta-analysis of field studies. *Soil Biol. Biochem.* 127 71–78. 10.1016/j.soilbio.2018.08.024

[B81] Pagan J. Child J. Scowcroft W. Gibson A. (1975). Nitrogen fixation by Rhizobium cultured on a defined medium. *Nature* 256 406–407. 10.1038/256406a0

[B82] Pajares S. Bohannan B. J. (2016). Ecology of nitrogen fixing, nitrifying, and denitrifying microorganisms in tropical forest soils. *Front. Microbiol.* 7:1045. 10.3389/fmicb.2016.01045 27468277 PMC4932190

[B83] Pastorelli R. Vignozzi N. Landi S. Piccolo R. Orsini R. Seddaiu G. (2013). Consequences on macroporosity and bacterial diversity of adopting a no-tillage farming system in a clayish soil of Central Italy. *Soil Biol. Biochem.* 66 78–93. 10.1016/j.soilbio.2013.06.015

[B84] Patkowska E. Konopiński M. (2013). Effect of cover crops on the microorganisms communities in the soil under scorzonera cultivation. *Plant Soil Environ.* 59 460–464. 10.17221/408/2013-pse

[B85] Pepe-Ranney C. Campbell A. N. Koechli C. N. Berthrong S. Buckley D. H. (2016). Unearthing the ecology of soil microorganisms using a high resolution DNA-SIP approach to explore cellulose and xylose metabolism in soil. *Front. Microbiol.* 7:703. 10.3389/fmicb.2016.00703 27242725 PMC4867679

[B86] Peres-Neto P. R. Legendre P. Dray S. Borcard D. (2006). Variation partitioning of species data matrices: estimation and comparison of fractions. *Ecology* 87 2614–2625. 10.1890/0012-9658(2006)87[2614:vposdm]2.0.co;2 17089669

[B87] Philippot L. Spor A. Hénault C. Bru D. Bizouard F. Jones C. M. (2013). Loss in microbial diversity affects nitrogen cycling in soil. *ISME J.* 7 1609–1619. 10.1038/ismej.2013.34 23466702 PMC3721106

[B88] Pichinoty F. Bigliardi-Rouvier J. Mandel M. Greenway B. Méténier G. Garcia J.-L. (1976). The isolation and properties of a denitrifying bacterium of the genus Flavobacterium. *Antonie Van Leeuwenhoek* 42 349–354. 10.1007/BF00394134 1086648

[B89] Postma J. Van Veen J. Walter S. (1989). Influence of different initial soil moisture contents on the distribution and population dynamics of introduced Rhizobium leguminosarum biovar trifolii. *Soil Biol. Biochem.* 21 437–442. 10.1016/0038-0717(89)90156-9

[B90] Prasad S. Malav L. C. Choudhary J. Kannojiya S. Kundu M. Kumar S. (2021). “Soil microbiomes for healthy nutrient recycling,” in *Current Trends in Microbial Biotechnology for Sustainable Agriculture*, eds Yadav A. N. Singh J. Singh C. Yadav N. (Berlin: Springer), 1–21. 10.1007/978-981-15-6949-4_1

[B91] Ramachandra M. Crawford D. L. Hertel G. (1988). Characterization of an extracellular lignin peroxidase of the lignocellulolytic actinomycete Streptomyces viridosporus. *Appl. Environ. Microbiol.* 54 3057–3063. 10.1128/aem.54.12.3057-3063.1988 3223769 PMC204427

[B92] Ramirez K. S. Craine J. M. Fierer N. (2012). Consistent effects of nitrogen amendments on soil microbial communities and processes across biomes. *Glob. Chang. Biol.* 18 1918–1927. 10.1111/j.1365-2486.2012.02639.x

[B93] Ramirez K. S. Lauber C. L. Knight R. Bradford M. A. Fierer N. (2010). Consistent effects of nitrogen fertilization on soil bacterial communities in contrasting systems. *Ecology* 91 3463–3470. 10.1890/10-0426.1 21302816

[B94] Rasmann S. Kollner T. G. Degenhardt J. Hiltpold I. Toepfer S. Kuhlmann U. (2005). Recruitment of entomopathogenic nematodes by insect-damaged maize roots. *Nature* 434 732–737. 10.1038/nature03451 15815622

[B95] Rhine E. D. Mulvaney R. L. Pratt E. J. Sims G. K. (1998). Improving the Berthelot reaction for determining ammonium in soil extracts and water. *Soil Sci. Soc. Am. J.* 62, 473–480. 10.2136/sssaj1998.03615995006200020026x

[B96] Rognes T. Flouri T. Nichols B. Quince C. Mahé F. (2016). VSEARCH: a versatile open source tool for metagenomics. *PeerJ* 4:e2584. 10.7717/peerj.2584 27781170 PMC5075697

[B97] Romdhane S. Spor A. Busset H. Falchetto L. Martin J. Bizouard F. (2019). Cover crop management practices rather than composition of cover crop mixtures affect bacterial communities in no-till agroecosystems. *Front. Microbiol.* 10:1618. 10.3389/fmicb.2019.01618 31338089 PMC6629898

[B98] Rong Q. Cai Y. Chen B. Yue W. Tan Q. (2017). An enhanced export coefficient based optimization model for supporting agricultural nonpoint source pollution mitigation under uncertainty. *Sci. Total Environ.* 580 1351–1362. 10.1016/j.scitotenv.2016.12.099 28017417

[B99] Rousk J. Baath E. Brookes P. C. Lauber C. L. Lozupone C. Caporaso J. G. (2010). Soil bacterial and fungal communities across a pH gradient in an arable soil. *ISME J.* 4 1340–1351. 10.1038/ismej.2010.58 20445636

[B100] Rousk J. Brookes P. C. Baath E. (2009). Contrasting soil pH effects on fungal and bacterial growth suggest functional redundancy in carbon mineralization. *Appl. Environ. Microbiol.* 75 1589–1596. 10.1128/AEM.02775-08 19151179 PMC2655475

[B101] Schloss P. D. (2020). Reintroducing mothur: 10 years later. *Appl. Environ. Microbiol.* 86 e02343–19. 10.1128/AEM.02343-19 31704678 PMC6952234

[B102] Schloss P. D. Westcott S. L. Ryabin T. Hall J. R. Hartmann M. Hollister E. B. (2009). Introducing mothur: open-source, platform-independent, community-supported software for describing and comparing microbial communities. *Appl. Environ. Microbiol.* 75 7537–7541. 10.1128/AEM.01541-09 19801464 PMC2786419

[B103] Schmidt R. Gravuer K. Bossange A. V. Mitchell J. Scow K. (2018). Long-term use of cover crops and no-till shift soil microbial community life strategies in agricultural soil. *PLoS One* 13:e0192953. 10.1371/journal.pone.0192953 29447262 PMC5814021

[B104] Schramm A. de Beer D. van den Heuvel J. C. Ottengraf S. Amann R. (1999). Microscale distribution of populations and activities of Nitrosospira and Nitrospira spp. along a macroscale gradient in a nitrifying bioreactor: quantification by in situ hybridization and the use of microsensors. *Appl. Environ. Microbiol.* 65 3690–3696. 10.1128/AEM.65.8.3690-3696.1999 10427067 PMC91552

[B105] Simmons B. L. Coleman D. C. (2008). Microbial community response to transition from conventional to conservation tillage in cotton fields. *Appl. Soil Ecol.* 40 518–528. 10.1016/j.apsoil.2008.08.003

[B106] Simon J. Klotz M. G. (2013). Diversity and evolution of bioenergetic systems involved in microbial nitrogen compound transformations. *Biochim. Biophys. Acta Bioenergetics* 1827 114–135. 10.1016/j.bbabio.2012.07.005 22842521

[B107] Songjuan G. Weidong C. Guopeng Z. (2021). Bacterial communities in paddy soils changed by milk vetch as green manure: a study conducted across six provinces in South China. *Pedosphere* 31 521–530. 10.1016/s1002-0160(21)60002-4

[B108] Spieck E. Sass K. Keuter S. Hirschmann S. Spohn M. Indenbirken D. (2020). Defining culture conditions for the hidden nitrite-oxidizing bacterium Nitrolancea. *Front. Microbiol.* 11:1522. 10.3389/fmicb.2020.01522 32849321 PMC7365893

[B109] Sun R. Guo X. Wang D. Chu H. (2015). Effects of long-term application of chemical and organic fertilizers on the abundance of microbial communities involved in the nitrogen cycle. *Appl. Soil Ecol.* 95 171–178. 10.1139/cjm-2018-0683 30901528

[B110] Sun Z. Lv Y. Liu Y. Ren R. (2016). Removal of nitrogen by heterotrophic nitrification-aerobic denitrification of a novel metal resistant bacterium Cupriavidus sp. S1. *Bioresour. Technol.* 220 142–150. 10.1016/j.biortech.2016.07.110 27566522

[B111] Tanaka J. S. Huba G. J. (1985). A fit index for covariance structure models under arbitrary GLS estimation. *Br. J. Math. Stat. Psychol.* 38 197–201. 10.1111/j.2044-8317.1985.tb00834.x

[B112] Tatti E. Goyer C. Burton D. L. Wertz S. Zebarth B. J. Chantigny M. (2015). Tillage management and seasonal effects on denitrifier community abundance, gene expression and structure over winter. *Microb. Ecol.* 70 795–808. 10.1007/s00248-015-0591-x 25851442

[B113] Tian J. Dungait J. A. Lu X. Yang Y. Hartley I. P. Zhang W. (2019). Long-term nitrogen addition modifies microbial composition and functions for slow carbon cycling and increased sequestration in tropical forest soil. *Glob. Chang. Biol.* 25 3267–3281. 10.1111/gcb.14750 31273887

[B114] Timonen S. Finlay R. D. Olsson S. Söderström B. (1996). Dynamics of phosphorus translocation in intact ectomycorrhizal systems: non-destructive monitoring using a β-scanner. *FEMS Microbiol. Ecol.* 19 171–180. 10.1016/0168-6496(96)00002-5

[B115] Toda M. Uchida Y. (2017). Long-term use of green manure legume and chemical fertiliser affect soil bacterial community structures but not the rate of soil nitrate decrease when excess carbon and nitrogen are applied. *Soil Res.* 55 524–533. 10.1071/sr17109

[B116] Trevors J. J. (1998). Bacterial biodiversity in soil with an emphasis on chemically-contaminated soils. *Water Air Soil Pollut.* 101 45–67.

[B117] Urakami T. Sasaki J. Suzuki K.-I. Komagata K. (1995). Characterization and Description of Hyphomicrobium denitrificans sp. nov. *Int. J. Syst. Evol. Microbiol.* 45 528–532. 10.1099/00207713-45-3-528

[B118] Verbaendert I. Boon N. De Vos P. Heylen K. (2011). Denitrification is a common feature among members of the genus Bacillus. *Syst. Appl. Microbiol.* 34 385–391. 10.1016/j.syapm.2011.02.003 21530125

[B119] Vukicevich E. Lowery T. Bowen P. Úrbez-Torres J. R. Hart M. (2016). Cover crops to increase soil microbial diversity and mitigate decline in perennial agriculture. A review. *Agron. Sustain. Dev.* 36 48.

[B120] Wang Q. Garrity G. M. Tiedje J. M. Cole J. R. (2007). Naive Bayesian classifier for rapid assignment of rRNA sequences into the new bacterial taxonomy. *Appl. Environ. Microbiol.* 73 5261–5267. 10.1128/AEM.00062-07 17586664 PMC1950982

[B121] Wang W. Yang M. Shen P. Zhang R. Qin X. Han J. (2019). Conservation tillage reduces nitrous oxide emissions by regulating functional genes for ammonia oxidation and denitrification in a winter wheat ecosystem. *Soil Tillage Res.* 194:104347. 10.1016/j.still.2019.104347

[B122] Wang Y. Li C. Tu C. Hoyt G. D. DeForest J. L. Hu S. (2017). Long-term no-tillage and organic input management enhanced the diversity and stability of soil microbial community. *Sci. Total Environ.* 609 341–347. 10.1016/j.scitotenv.2017.07.053 28753509

[B123] Wang Z. Liu L. Chen Q. Wen X. Liao Y. (2016). Conservation tillage increases soil bacterial diversity in the dryland of northern China. *Agron. Sustain. Dev.* 36 1–9.

[B124] Warton D. I. Wright S. T. Wang Y. (2012). Distance-based multivariate analyses confound location and dispersion effects. *Methods Ecol. Evol.* 3 89–101. 10.1186/s40168-019-0659-9 30935409 PMC6444669

[B125] Wu Y. Lu L. Wang B. Lin X. Zhu J. Cai Z. (2011). Long-term field fertilization significantly alters community structure of ammonia-oxidizing bacteria rather than archaea in a paddy soil. *Soil Sci. Soc. Am. J.* 75 1431–1439. 10.2136/sssaj2010.0434

[B126] Xi H. Shen J. Qu Z. Yang D. Liu S. Nie X. (2019). Effects of long-term cotton continuous cropping on soil microbiome. *Sci. Rep.* 9 1–11. 10.1038/s41598-019-54771-1 31797982 PMC6892916

[B127] Zahran H. H. (1999). Rhizobium-legume symbiosis and nitrogen fixation under severe conditions and in an arid climate. *Microbiol. Mol. Biol. Rev.* 63 968–989. 10.1128/MMBR.63.4.968-989.1999 10585971 PMC98982

[B128] Zeng J. Liu X. Song L. Lin X. Zhang H. Shen C. (2016). Nitrogen fertilization directly affects soil bacterial diversity and indirectly affects bacterial community composition. *Soil Biol. Biochem.* 92 41–49. 10.1038/ismej.2011.159 22134642 PMC3329107

[B129] Zhang C. Song Z. Zhuang D. Wang J. Xie S. Liu G. (2019). Urea fertilization decreases soil bacterial diversity, but improves microbial biomass, respiration, and N-cycling potential in a semiarid grassland. *Biol. Fertil. Soils* 55 229–242. 10.1007/s00374-019-01344-z

[B130] Zhang H. Zhao Z. Li S. Chen S. Huang T. Li N. (2019). Nitrogen removal by mix-cultured aerobic denitrifying bacteria isolated by ultrasound: performance, co-occurrence pattern and wastewater treatment. *Chem. Eng. J.* 372 26–36. 10.1016/j.cej.2019.04.114

[B131] Zhou J. Jiang X. Wei D. Zhao B. Ma M. Chen S. (2017). Consistent effects of nitrogen fertilization on soil bacterial communities in black soils for two crop seasons in China. *Sci. Rep.* 7:3267. 10.1038/s41598-017-03539-6 28607352 PMC5468298

