# Supplementary figures and images for: Variation in Bacterial Community Structure Under Long-Term Fertilization, Tillage, and Cover Cropping in Continuous Cotton Production
Source: Front Microbiol. 2022 Apr 4;13:847005. doi: 10.3389/fmicb.2022.847005 (PMC9015707; doi:10.3389/fmicb.2022.847005)

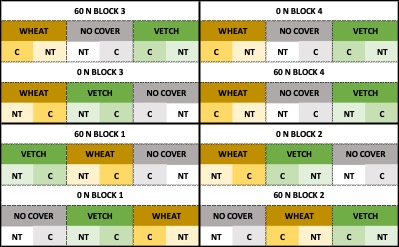

Supplement: Supplementary Figure 1 — Field design (RCBD with split-split plot). The plots are 40′ × 26.6′ with 20′ alleys. Total average with alleys 4.5 ac, main plot: nitrogen fertilization rate (0, 60 lb N/ac), Subplot: cover crop, no-cover, hairy vetch, crimson clover, wheat. Sub-sub plot: Tillage; C, conventional till; NT, no-till. [file Image_1.JPEG]

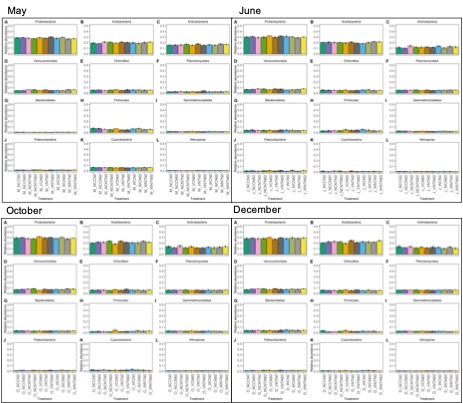

Supplement: Supplementary Figure 2 — The relative abundance of bacterial phyla, including Proteobacteria (A), Acidobacteria (B), Actinobacteria (C), Verrucomicrobia (D), Choroflexi (E), Planctomycetes (F), Bacteroidetes (G), Firmicutes (H), Gemmatimonadetes (I), Patescibacteria (J), Cynaobacteria (K), Nitrospirae (L) across treatments collected in May, June, October, and December. [file Image_2.JPEG]
